# Experiences of frontline nursing staff on workplace safety and occupational health hazards in two psychiatric hospitals in Ghana

**DOI:** 10.1186/s12889-018-5620-5

**Published:** 2018-06-06

**Authors:** Robert Kaba Alhassan, Kwabena Adu Poku

**Affiliations:** 1 Department of Public Health Nursing, School of Nursing and Midwifery University of Health and Allied Sciences, Ho. PMB 31, Volta Region Ho, Ghana; 2 Formerly of Department of Public Administration and Health Services Management, University of Ghana Business School, University of Ghana Legon. LG 25, Accra, Legon, Ghana

**Keywords:** Occupational health, Safety, Psychiatric hospitals, Nurses, Nurse-assistants, Accra, Pantang, Ghana

## Abstract

**Background:**

Psychiatric hospitals need safe working environments to promote productivity at the workplace. Even though occupational health and safety is not completely new to the corporate society, its scope is largely limited to the manufacturing/processing industries which are perceived to pose greater dangers to workers than the health sector. This paper sought to explore the experiences of frontline nursing personnel on the occupational health and safety conditions in two psychiatric hospitals in Ghana.

**Methods:**

This is an exploratory cross-sectional study among 296 nurses and nurse-assistants in Accra (*n* = 164) and Pantang (*n* = 132) psychiatric hospitals using the proportional stratified random sampling technique. Multivariate Ordinary Least Squares (OLS) regression test was conducted to ascertain the determinants of staff exposure to occupational health hazards and the frequency of exposure to these occupational health hazards on daily basis.

**Results:**

Knowledge levels on occupational health hazards was high in Accra and Pantang psychiatric hospitals (i.e. 92 and 81% respectively), but barely 44% of the 296 interviewed staff in the two hospitals said they reported their most recent exposure to an occupational health hazard to hospital management. It was found that staff who worked for more years on the ward had higher likelihood of exposure to occupational health hazards than those who worked for lesser years (*p* = 0.002). The category of occupational health hazards reported most were the physical health hazards. Psychosocial hazards were the least reported health hazards. Frequency of exposure to occupational health hazards on daily basis was positively associated with work schedules of staff particularly, staff on routine day schedule (Coef = 4.49, *p* = 0.011) and those who alternated between day and night schedules (Coef = 4.48, *p* = 0.010).

**Conclusion:**

Occupational health and safety conditions in the two hospitals were found to be generally poor. Even though majority of the staff knew about occupational health and safety, less than half of them reported exposure to workplace health hazards. Key stakeholders such as the Ministry of Health in collaboration with the Mental Health Authority should intensify efforts towards effective enforcement of existing policies on safety in healthcare institutions, particularly psychiatric hospitals where exposure to occupational health hazards is more prevalent.

**Electronic supplementary material:**

The online version of this article (10.1186/s12889-018-5620-5) contains supplementary material, which is available to authorized users.

## Background

Every organization needs healthy and safe human resource base for its performance and general productivity. The health sector is no exception given that it is made up of diverse professional groups that are exposed to occupational health hazards at varying degrees. Even though the concept of occupational health and safety is not new to the corporate society, its scope until now was limited to only the manufacturing and processing industries which were perceived to pose greater dangers and havoc to workers. The service industries, including the health sector, were completely relegated to the background, especially in developing countries [1].

The International Labour Organization (ILO) and World Health Organization (WHO) Joint Committee on Occupational Health and Safety (JCOHS) in 1995 indicated that occupational health and safety in every work environment entails the promotion and maintenance of the highest degree of physical, mental and social well-being of workers in all occupations [2]. Sadleir [3] specified that occupational health and safety is that aspect of environmental health which entails the interaction between the workplace and the health of the worker. Occupational health and safety is critical for every health system because the health sector is highly labour intensive with health workers constituting a vital input factor in the health service production process.

Moreover, OHS is particularly important in resource poor settings because of limited logistics in many healthcare facilities, particularly psychiatric hospitals where working conditions are worst [4]. These poor working conditions usually compromise health and safety conditions in these healthcare facilities. The situation is often times aggravated by the poor legal foundations on OHS in some countries where physical and psychological violence against healthcare workers is implied to be part of their work [5].

This study became necessary because, most work related health hazards in developing settings such as Ghana go unreported [6, 7] usually due to ignorance on OHS and poor hospital management systems, particularly in psychiatric hospitals. Moreover, in many countries including Ghana, there are limited scientific publications on occupational health and safety in psychiatric hospitals [8–10]. Nonetheless, within the health sector psychiatric hospitals are work environments where employees are most at risk of exposure to occupational health hazards.

In a large survey conducted among 323 clinical staff in a public psychiatric hospital, Kelly et al. [8] found that nearly 70% of the staff had experienced physical assault in the last 12 months. Kelly et al. [8] observed that staff well-being in terms of depression, anger, physical health and safety were adversely affected by conflicts with other staff members and by individual response to social conflict and assault. In similar studies by Cornaggia et al. [9] and Kelly et al. [10] in different countries, it was found that about two thirds of staff working in psychiatric hospitals have experienced one form of occupational health hazard or the other.

In resource poor countries such as Ghana, the situation is assumed to be more given the limited human and material health resources needed to reduce the risk of exposure to these health hazards. In Ghana, existing laws on occupational health and safety are obsolete and perhaps these laws are not addressing emerging workplace challenges in the health sector. For instance, workplace safety standards in Ghana spelt out in the workman compensation law (PNDCL 187, 1987) are not being executed to the latter due to poor infrastructure and inadequate human resources at the workplace.

As part of efforts towards improving health and safety conditions in psychiatric hospitals in particular, the Mental Health Authority (MHA) act (Act 846, 2012) was passed albeit its full implementation remains a challenge. The MHA (Act 846, 2012) is currently not backed by a Legislative Instrument (LI) to compel government budgetary allocation in the planning and delivery of mental health services. Consequently the MHA is unable to carry out its full mandate including creating conducive environment for the safety of patients and staff, largely due to financial constraints.

This paper sought to explore experiences of nurses and nurse-assistants working in psychiatric hospitals on OHS in two psychiatric hospitals in Ghana. Key research objectives include ascertaining the knowledge and awareness of staff on OHS; determining the types of occupational health hazards, and how they are reported by staff.

## Methods

### Research design

The study is an exploratory cross-sectional study among nursing staff in two out of the three psychiatric hospitals in Ghana. The cross-sectional design was considered because it promotes attainment of larger sample size and generalizability to study populations. The design also enables the researchers to describe and examine associations between key variables of interest [11].

One of the key variables of interest in the study design was the frequency of exposure to occupational hazards where respondents were asked to give number times within the day that they experienced occupational health hazards. Due to potential recall bias, these responses were analyzed on daily averages because many respondents could not give exact number of times of exposure especially among staff who did not consider some occurrences at the workplace as occupational health hazards.

### Study population

There are only three psychiatric hospitals in Ghana serving a population of over 29 million. This study was conducted in two out of the three psychiatric hospitals. The two selected hospitals were Accra and Pantang psychiatric hospitals both located in the Greater Accra Region (GAR) of Ghana. The two psychiatric hospitals were purposively selected because they are currently the only healthcare facilities dedicated to psychiatric care in the GAR and since the focus of the research was on urban setting, these two hospitals were selected. Moreover, the two selected hospitals employ over 80% of psychiatric workforce in Ghana and also renders the major referral psychiatric services in the country. The third psychiatric hospital (Ankaful Psychiatric Hospital) is in the Central Region.

Accra Psychiatric Hospital (APH) is a specialized health facility that is responsible for the welfare, training, rehabilitation and treatment of the mentally ill [12]. As at the time of conducting this study in March, 2010, APH had a bed capacity of six hundred (600) patients at any given time with a daily in-patient population averaging between 1100 and 1300 [13]. There are 21 wards in the APH excluding the Out Patients Department (OPD). The total population of nurses and nurse-assistants then was 354. Professional nurses were 192 and nurses-assistants were 162 [13].

Pantang Psychiatric Hospital (PPH) on the other hand was established in 1975 and it is the youngest among the three psychiatric hospitals. It is located in the Ga East District of the GAR. PPH is also a specialized hospital that is responsible for treating mentally ill patients. The hospital has a nurses’ training school that trains psychiatric nurses and also gives affiliation training to nursing and medical students on psychiatry. The bed capacity is 200 but the in-patient population as at the time of conducting this study was 372 patients with a daily OPD attendance of approximately 73 patients [14].

The population of professional nurses in PPH as at February, 2010 was 198 and the nurse-assistants were 95, making a total of 293. These health care personnel work in ten (10) different wards: seven (7) wards for male patients and three (3) wards for female patients and the OPD [14].

### Sampling procedure

As indicated earlier, the two psychiatric hospitals were purposively selected because they are currently the only healthcare facilities dedicated to psychiatric care in the GAR. At the respondents’ level, proportional stratified random sampling technique was used. The aim was to promote equal representation of the various categories of nurses working in the sampled psychiatric hospitals. The stratified random sampling involved creating two main strata, namely, professional nurses and nurse-assistants in the two hospitals.

Quota system was further employed to allocate 60% of the study sample to professional nurses and the remaining 40% to nurse-assistants since the population of professional nurses was larger than the nurse-assistants in the two hospitals. Triangulation of the various sampling methods was meant to reduce bias and promote representativeness of the study sample. Individual respondents within the proportioned strata were subsequently sampled by simple random sampling.

For the purposes of this study, professional nurses include registered mental health nurses, registered general nurses, and registered community nurses. Nurse-assistants as used in this study include Nurse Assistant Clinical (NAC) and Nurse Assistant Preventative (NAP). Other non-professional nurse categories are Health Extension Workers (HEW), Ward Orderlies (WO) and Health Aids (HAs). Nursing personnel on post retirement contract and student nurses were excluded from the study because the focus was on experiences of full time staff on workplace safety and occupational health hazards.

### Sample size

Sample size for the two purposively selected facilities was based on the Krejcie and Morgan [15] statistical tool for determining sample size based on known populations. This statistical tool gives the recommended sample size for a finite population at the 95% level of confidence.

Krejcie and Morgan [15] stated that with a population size of 650, for example, the representative sample size should be at least 242 and since the population size for the two selected hospitals was 647, a sample size of 350 for both hospitals was deemed representative and generalizable to the whole population. The quota system sampling was used to allocate 60% (210) of the total sample size (*n* = 350) to APH and the remaining 40% (140) allocated to PPH. Out of the 350 participants earmarked for the study, 296 of them responded and returned completed questionnaires, representing 85% return rate.

### Data collection instrument

#### Process of tool development

The instrument used in the study was mainly structured questionnaires where staff filled in the questionnaires themselves and returned later. To promote a high return rate, some of the staff were assisted to fill in the questionnaire but these were not structured interviews. The questionnaire was developed following a rigorous review of relevant literature on safety in healthcare institutions. Recurrent themes were later synthesized to validate and inform content of the questionnaire. Moreover, exploratory individual interviews were conducted among professional nurses and nurse-assistants. Responses from these interviews revealed critical topic areas for development of the questions using the inductive approach. In sum, development of the data collection tool was guided by the reviewed literature and validated based on responses from the exploratory staff interviews. The piloting and content review processes by co-author and other peers were also employed to validate the tool before implementation.

### Items included in the data collection tool

The data collection tool was structured in eight (8) sections, namely: SECTION A (respondent’s biographic data), SECTION B (respondent’s work history), SECTION C (respondent’s knowledge of occupational health and safety), SECTION D (respondent’s experience of occupational health hazards), SECTION E (responsibility for work related health hazards), SECTION F (respondent’s reporting system of exposure to occupational health hazards), SECTION G (respondent’s perception of safety conditions of psychiatric hospitals), SECTION H (respondent’s suggestions for improving safety in psychiatric hospitals). On a whole, the questionnaire had 31 content questions excluding questions on demographic characteristics (i.e. age and gender) and work history (see Additional file 1).

Nine (9) questions out of the 31 content questions were focused on staff perception of safety conditions of psychiatric hospitals. These nine questions were structured in a five-point Likert scale. The five point Likert scale was designed as follows: 1 = “very bad”, 2 = “bad”, 3 = “average”, 4 = “good” and 5 = “very good”. The rest of the content questions were close (e.g. Yes or No) and open ended questions.

### Exploration of administrative records

Besides the administration of questionnaires to staff, existing administrative records in the two sampled facilities were reviewed. This approach was meant to independently ascertain the number of documented cases of exposure to occupational health hazards. Also, at the time of conducting this study there were not many available scientific publications on the topic area, particularly in the context of psychiatric hospitals. In view of this, there was the need to complement the questionnaires administration to review of existing hospital archival records on incidence of exposure to occupational health hazards in the two hospitals. Incidence registers were reviewed in eight (8) out of the twenty one (21) wards in Accra psychiatric hospital and eight (8) out of the ten (10) wards in Pantang psychiatric hospital throughout the period of the data collection.

### Reliability and internal validity of data collection tool

To ensure internal validity, all questions were informed by the research objectives and the reviewed literature. Moreover, Cronbach’s alpha (α) was conducted to check for scale reliability of the nine (9) five-point Likert scale questions on perceived workplace safety factors. The Cronbach’s alpha (α) test was found to be above the 0.70 rule of thumb desired for scale reliability. All the nine Likert Scale questions assumed one underlying dimension as follows: 1 = “very bad”, 2 = “bad”, 3 = “average”, 4 = “good” and 5 = “very good”. Piloting of the data collection tool also offered the researchers the opportunity to improve the quality of the questions and thus promote its reliability and internal validity.

### External validity of the study

Involvement of the different cadres of nursing staff from the two psychiatric hospitals was meant to promote external validity of the study findings. Moreover, sampling the two largest psychiatric hospitals in Ghana guarantees external validity and generalizability of the findings because, over 80% of nursing staff working in psychiatric hospitals in Ghana have been covered. In light of this, it is most likely that views expressed in this study largely represent the general perceptions of healthcare personnel in psychiatric hospitals in Ghana.

### Data collection schedule and mode

The schedule for data collection started with permission and administrative approval from the selected health facilities for the study. This was done a week prior to the commencement of the data collection. Afterwards, questionnaires were administered to sampled respondents of the two hospitals.

### Piloting

Piloting was done with ten questionnaires in two district hospitals in the GAR. The pretesting was meant to check for consistency, relevance and comprehensiveness of the questions. The pretest responses were desirable and content change was not needed. However, few typographical errors were identified and corrected.

### Role of research assistants

The questionnaires distribution was done with the assistance of two trained research assistants. The research assistants’ role included visiting the two psychiatric hospitals, and administering and retrieving questionnaires from respondents who met the inclusion criteria for the study. Administration of questionnaires was done simultaneously in the two hospitals to avoid sensitization of respondents who will be contacted in the other hospital later. The questionnaire administration took approximately fourteen (14) days, including mob-up days for staff who could not complete their questionnaires on the day of visit.

### Operational definition of terms

To avoid ambiguity in interpretation of the terms by research assistants to respondents, operational definitions were clearly stated to accompany administration of structured questionnaires. Operational definition of terms are explained in the endnote.^1^

### Data analysis

The data collected from the surveys was analyzed with the Statistical Package for Social Sciences (SPSS) version 22.0. Relevant frequency distribution tables were generated for the type of occupational health hazards experienced by the frontline health workers. Multivariate Ordinary Least Squares (OLS) regression analysis was performed to determine the associations between individual characteristics and work schedules of health staff and their frequency of exposure to occupational health hazards on daily basis. Prior to the regression analysis, multicollinearity diagnosis was conducted for all the independent variables of interest and none had a Variance Inflation Factor (VIF) up to 10.0, required for inclusion in the regression model.

The first dependent variable was “Exposure to occupational health hazards”, dichotomized into 0 = “No” and 1 = “Yes” while the second dependent variable was “Frequency of exposure” which is a continuous variable in terms of number of times of exposure to occupational health hazards on daily basis.

The explanatory/independent variables of interest were professional category (Professional Nurse or Other category of staff), and Work Schedules of staff (Day shift, Day and night, Night only). Other variables controlled for in the regression model were: staff age, gender (male or female) and number of years of work experience.

Moreover, all five-point Likert scale items were dichotomized into two outcomes by combining “very bad” and “bad” into “Bad”. “Very good”, “good” and “Average” were combined into “Good”. Test for statistical significance was set at 95%, thus *p*-values less than 0.05 were deemed statistically significant as appropriate.

## Results

### Demographic data and work history of respondents

Two hundred and ten (210) questionnaires were administered in the APH and 164 of them were retrieved with complete data, representing a return rate of 78%. However, the return rate at the PPH was 96% out of 140 administered questionnaires. The total valid responses from the two hospitals was thus, 296. The field data showed that females dominated in both hospitals, representing 65%. Professional nurses also dominated, representing 60% while nurse-assistants constituted 39% (see Table 1). On the work schedules of respondents, it was found that staff who alternated between day and night work shifts dominated 145 (49%); 142 (48%) worked mainly on day shift; three staff (1%) indicated they run permanent night shift. Thus, it was observed that majority of the staff (97%) either alternated day and night shifts or worked solely on day shift. Approximately 50% of the staff worked for 2 years or more and 49% worked for 1 year or less (see Table 1).

**Table 1:** Demographic and professional characteristics of respondents in APH and PPH (*n* = 296)

Characteristics	Statistic	
	Freq.	%^c^
Gender		
Male	105	35
Female	191	65
Age		
≤ 30 years	170	57
31–60 years	64	22
Missing data^d^	62	21
Professional Category		
Professional Nurses	179	60
Nurse-Assistants	115	39
Missing data^d^	2	1
Work schedules		
Alternate between day and night shifts	145	49
Permanent day shift only	142	48
Permanent night shift only	3	1
Missing data^d^	6	2
Work Experience		
More Experienced^a^	148	50
Less Experienced^b^	145	49
Missing data^d^	3	1

Source: Field Data (2010)

Legend: *APH* Accra Psychiatric Hospital, *PPH* Pantang Psychiatric Hospital, *n* sample size

^a^Modeled in this study to mean workers who worked for 2 years or more

^b^Modeled in this study to mean Workers who worked for 1 year or less

^c^All percentages have been rounded up to the nearest decimal point

^d^Number of respondents who did not answer those questions

### Staff awareness and experiences of occupational health hazards

Out of the 296 valid responses from the two hospitals, it was found that 87% of them said they are aware of occupational health hazards at their workplaces and that working in a psychiatric hospital was more risky than other healthcare facilities. In a follow-up open ended question, the responses suggest that the perceived high risk of staff exposure was because the two hospitals are major referral psychiatric hospitals. The overwhelming inpatient and outpatient attendance against poor staff strength, in the opinion of staff, posed significant challenge coupled with overcrowding which has dire consequences on occupational health and safety of personnel in the two hospitals.

Out of the 258 staff who were aware of OHHs, it was found that the predominant category of OHH experienced by the health workers was physical health hazards (53%) followed by biological (20%) and psychosocial (17%) health hazards (see Table 2). Specific physical hazards experienced included physical assault and battery by clients at the acute stage of their mental health condition. Verbal abuse, work related stress, and superior relations with subordinates were mentioned by respondents as major psychosocial health hazards often experienced at the workplace. Additionally, exposure to viral, bacterial or fungal infections were the predominant biological hazards reported due to exposure to body fluids of clients without adequate personal protective wears.

**Table 2:** Responses on awareness of occupational health and safety in APH and PPH (*n* = 296)

Variables	Statistic	
	Freq.	%^a^
Awareness of OHH		
Yes	258	87
No	35	12
Missing data^b^	3	1
Predominant source of information on OHH		
Seminars/workshop	90	30
Friends	13	4
Media	35	12
School lectures	74	25
Co-workers	17	6
Other	17	6
Missing data^b^	50	17
Predominant occupational hazards experienced		
Biological Health Hazards	52	20
Physical Health Hazards	137	53
Psychosocial Health Hazards	44	17
Missing data^b^	26	10
Episodes of exposure to occupational hazards		
One – four episodes a day	65	22
Five episodes a day	14	5
More than five episodes a day	36	12
More than ten episodes a day	27	9
Cannot remember	104	35
Missing data^b^	50	17

Source: Field Data (2010)

Legend: *APH* Accra Psychiatric Hospital, *PPH* Pantang Psychiatric Hospital, *OHH* Occupational Health Hazard, *n* sample size

^a^All percentages have been rounded up to the nearest decimal point

^b^Number of respondents who did not answer those questions

The pre-dominant reasons cited by victims of these exposures were overcrowding and workload. Out of the 296 staff who completed the questionnaires it was found that 22% experience between one to four episodes of OHHs in a day in terms of frequency of exposure; 5% experience at least five episodes a day; 12% said more than five episodes a day; 9% said more than ten a day; 35% said they could not remember the number and 17% of the staff did not answer that question (see Table 2).

Quite a number of the participants got information on OHHs from formal sources such as seminars/workshops (30%) and pre-service education lectures (25%). Other information sources mentioned by the staff were: co-workers, mass media and friends (see Table 2).

### Reporting of most recent experienced OHHs to hospital management

**Fig. 1:**
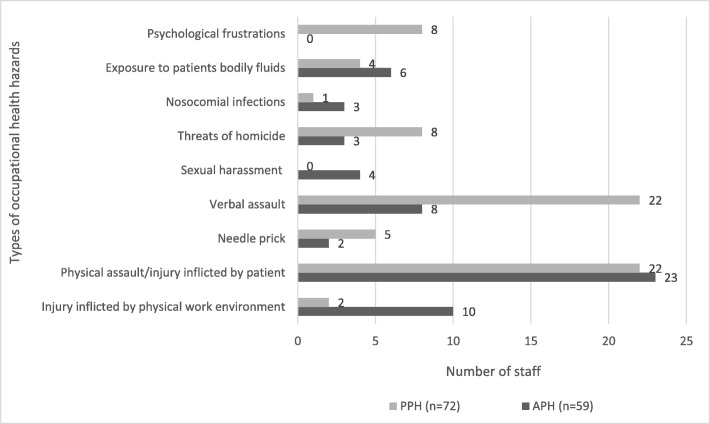
Most recent experienced occupational health hazards that were reported to hospital management by staff (*n* = 131). Legend: Source: Field Data (2010); PPH (Pantang Psychiatric Hospital); APH (Accra Psychiatric Hospital) n (Sample size)

Out of 296 staff who completed the questionnaires it was observed that 131 (44%) of them said they reported their most recent exposure to one form of OHH or the other to hospital management. Out of the 131 staff who reported their most recent exposures, 45(34%) of them reported physical assault/injury afflicted by patient including; 28(21%) reported verbal assault by patients; and 12(9%) reported injury inflicted by physical work environment. The remaining reported cases were needle stick pricks and sexual harassment (including attempted rape), and the rest were nosocomial infections (also called hospital acquired infections) due to poor sanitary conditions in the ward. Other reported OHHs were threats of homicide and tearing of uniform by patient; contact with body fluids of HIV/AIDS positive patient in the ward (see Fig. 1).

Follow-up open ended question was asked on why health workers refused to report exposure to OHHs and the responses included: “*problem was dealt with by myself*”, “*no channel to report work related health hazard*”, “*problem perceived as a minor problem*”, “*no measures would be taken*”, “*fear of reprimand by superiors*”, “*forgetfulness*”, “*no risk allowance paid*”, “*poor management of reported OHHs”*, “*blames from management for personal negligence at work*”.

### Findings on OHHs from hospital administrative records

**Fig. 2:**
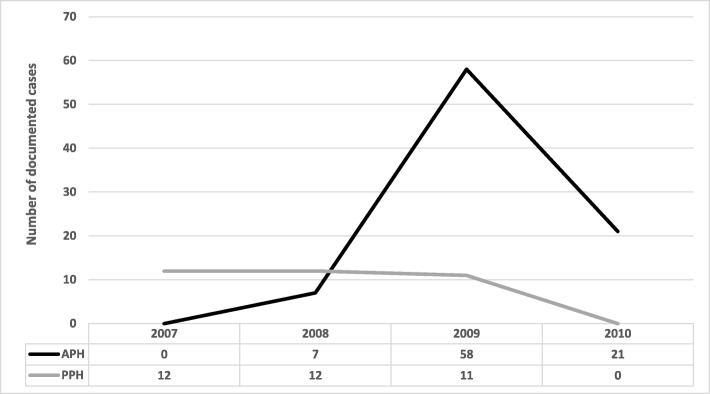
Administrative records on staff exposure to OHHs in APH and PPH (*n* = 121). Legend: Source: Field Data (2010); APH (Accra Psychiatric Hospital); PPH (Pantang Psychiatric Hospital); n (Sample size)

It was found that the total number of documented cases of OHHs over a three year period (2008–2010) in APH was 86 cases while in PPH, 35 cases were documented between 2007 and 2009. There were no available validated records for 2007 in APH and 2010 in PPH hence the absence of data in these years. Overall, the highest number of documented cases was in 2009 (69 cases) followed by 21 cases in 2010. In 2008, 19 cases were documented in both hospitals while the least documented cases were in 2007 (see Fig. 2). Perhaps these figures might be have been under-reported because of potential inconsistent/irregular documentation of these episodes of exposure to OHHs.

### Perception of respondents on safety conditions in their hospitals

**Fig. 3:**
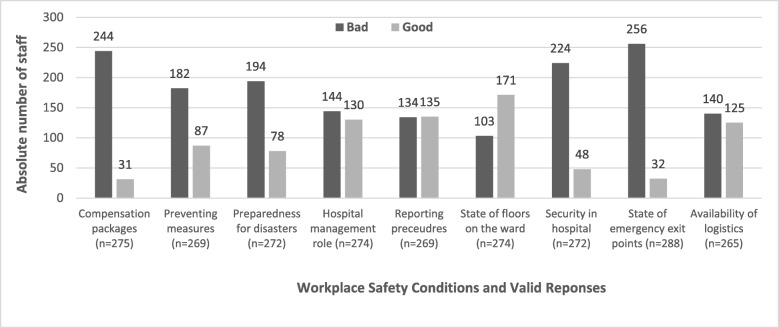
Perceived workplace conditions in APH and PPH. Legend: Source: Field Data (2010); APH (Accra Psychiatric Hospital); PPH (Pantang Psychiatric Hospital)

It was found that the number of valid responses for the different workplace conditions varied (see Fig. 3). For instance, out of the 265 valid responses on availability of logistics and material resources in the two hospitals, 53% rated that parameter as “bad” and the remaining 47% said it was “good”. On the state of emergency exit points on the wards, 87% out of 288 respondents rated this safety condition as “bad” and the remaining 13% rated it as: “good”. The security measures for workers of the two hospitals was rated by 224(82%) respondents as “bad” and 48 (18%) staff said it was “good”. Likewise, compensation packages for victims of OHHs; availability of preventive measures and hospital preparedness for disasters were rated by over 60% of the respondents as “bad”. Fifty percent (50%) of the staff indicated that the reporting procedures for exposure to OHHs in their hospitals was “bad” and the remaining 50% said it was “good”. The state of the hospital ward floors was described by 62% of the staff as “good” and 38% described the floors as “bad” (see Fig. 3).

### Determinants of exposure to occupational health hazards at the workplace

A multivariate regression was conducted to determine the factors associated with staff exposure to occupational health hazards. The dependent variables fitted in the regression model were “Exposure to occupational health hazards” (reported as a dichotomous variable, 0 = “No” and 1 = “Yes”) and “Frequency of exposure to occupational health hazards on daily basis” (reported as a count variable, number of times of exposure daily). The independent variables were: age of staff, gender, and years of work experience, work schedule and professional category (i.e. Nurse or Non-nurse).

It was found that age negatively correlates with the likelihood of a staff experiencing an occupational health hazard. Thus, elderly staff were less likely to experience OHHs than relatively younger staff (Coef. = − 0.16, *p* = 0.012) at 95% confidence level. However, the frequency of exposure to OHHs on daily basis had no significant association with the age of the staff. It was further observed that male staff were at higher risk of experiencing OHHs than female staff, albeit statistically not significant. Likewise, in terms of professional category, frequency of exposure to OHHs was less likely to occur among professional nurses than non-professional nurses even though the difference was not statistically significant.

In terms of work duty schedule and exposure to OHHs, staff who worked on day duty schedule only (Coef. = 4.49, *p* = 0.011), or alternating day and night schedules (Coef. = 4.83, *p* = 0.010) had a higher likelihood of exposure to OHHs than staff who worked mainly on night duty schedule. Counter intuitively, staff with more years of work experience had a higher chance of exposure to OHHs (Coef. = 0.07, *p* = 0.002) than those who relatively worked for less number of years (see Table 3).

**Table 3:** Determinants of staff exposure to occupational health and safety at the workplace

	Dependent variables							
	Exposure to OHH				Frequency of Exposure (Daily)			
Independent variables	Coef.^b^	95%	CI	*p*-value	Coef. ^b^	95%	CI	*p*-value^a^
Age	−0.16	−0.28	−0.03	0.012^a^	−0.43	−1.24	0.39	0.301
Gender								
Male	0.15	−0.00	0.30	0.052	−0.79	−1.80	0.22	0.124
Female	Ref			Ref	Ref			Ref
Profession								
Professional nurse	−0.01	−0.26	0.23	0.912	−1.39	−3.05	0.27	0.099
Other	Ref			Ref	Ref			Ref
Work schedule								
Day only	0.22	−0.29	0.73	0.400	4.49	1.05	7.93	0.011^a^
Day and night	0.28	−0.26	0.82	0.308	4.83	1.20	8.45	0.010^a^
Night only	Ref			Ref	Ref			Ref
Work experience	0.07	0.03	0.11	0.002^a^	0.15	−0.13	0.43	0.285

Source: Field Data (2010)

Legend: *APH* Accra Psychiatric Hospital, *OHH* Occupational Health Hazard, *OHS* Occupational Health and Safety, n Sample size, *CI* Confidence Interval

^a^Multivariate Ordinary Least Squares (OLS) regression analysis: p-values are statistically significant at 95% confidence level

^b^Ordinary Least Squares (OLS) regression coefficients

## Discussion

Empirical studies suggest that psychiatric healthcare facilities are increasingly becoming the most dangerous working environments within the health sector in many countries across the globe [16–20]. Resource poor countries such as Ghana are particularly the worst affected given the limited health infrastructure coupled with low health worker density per population of clients with mental health conditions.

In this study it was observed that, exposure of health workers to OHHs in the two sampled hospitals was high. It was also found that non-professional nurses (nurse-assistants) were more likely to experience exposure to OHHs than their professional counterparts. This observation confirms findings of previous studies that concluded that professional nurses were less likely to experience work related health hazards than lower level cadre of health workers [16, 21]. However, reasons for this variability were not statistically ascertained in this study.

Nonetheless, the observation could be attributed to the fact that nurse-assistants undergo relatively lesser number of years of training and might not have gained the needed professional expertise in adhering to occupational health and safety standards in psychiatric hospital environments. Perhaps this observation could also be attributed to the fact that in Ghana, there are no certificate level psychiatric nurses. Consequently, all the nurse-assistants working in these two psychiatric hospitals are either auxiliary nurses in general nursing or community health nursing and these cadre usually don’t have exhaustive training in psychiatric nursing comparable to registered professional mental health nurses which potentially poses higher risk to them and increases their chances of exposure to OHHs.

Since details on the determinants of exposure to OHHs were not explored in this current study, it is recommended that future studies should consider investigating the determinants of exposure to OHHs among professional and non-professional nursing personnel in the three psychiatric health hospitals in Ghana.

It was found there were no statistically significant differences in the exposure of male and female frontline nursing staff to OHHs in this study. Nonetheless, it is imperative future research investigations interrogate further the gender dynamics in exposure to OHHs with perhaps larger sample size in all the three psychiatric hospitals in Ghana.

Previous literature on OHHs in psychiatric hospitals in other countries found significant differences in the exposure of male and female health workers to OHHs [22, 23] which could be explained by differences in country contexts and methodological research approaches. For instance, Harnois and Gabriel [24] found that female frontline health workers were more likely to experience frequent exposures to workplace hazards in psychiatric healthcare facilities because they were disadvantaged in terms of self-defence against physical attacks by aggressive patients.

In similar studies conducted outside Ghana it was found that frontline female staff in psychiatric hospitals were less likely to report OHHs such as sexual harassment, including attempted rape and humiliating insults because they are deemed to be embarrassing and the victims could potentially be stigmatized by colleague workers [25].

In view of this research findings, it is recommended that education for frontline health workers be increased by the Mental Health Authority and other relevant stakeholders to help encourage victims to report exposures to OHHs. In addition, given that not many health workers had information on OHHs from formal sources in this study, it is recommended formal education (through in-service training workshops) are routinely organized to raise awareness on workplace safety in psychiatric hospitals.

In terms of the category of OHHs reported, physical health hazards were reported most by health workers in both hospitals. This observation contradicts studies by Carmel and Hunter [26] and Barron and Neuman [25] who concluded that psychosocial health hazards were the commonest occupational health hazards experienced by frontline psychiatric health staff. It was also found in this study that biological, psychosocial and chemical health hazards were minimally reported by the health workers in the two psychiatric hospitals. Albeit psychosocial health hazards were experienced more by health workers than physical health hazards, the former were minimally reported and documented by the hospitals as health hazard because of the misconception that such health hazards were “minor” and did not merit reporting [17, 24, 27–29].

Perhaps psychosocial health hazards such as insults by patients, spitting on nurses, verbal threats of homicide, unjust reprimand by superiors and other stressful events were glossed over as health hazards. The low reporting of psychosocial health hazards might be due to ignorance of these components of OHHs. Future studies may have to specifically focus on health workers’ perception of what constitutes an OHH.

On the precursors of exposure to occupational health hazards, most of the frontline health workers in this study attributed their exposure to OHHs to hospital management’s fault or inefficiency. Some respondents attributed the poor workplace safety situation to central government and the Ministry of Health for the inability to mobilize adequate health resources to promote occupational health and safety in psychiatric hospitals.

Another revelation was that the reporting system of work related health hazards in the two hospitals was low compared to studies on developed countries [30–34]. For instance, less than 60% of the respondents in both hospitals said they ever reported a work related health hazard to hospital management. This observation confirms the argument by Richards [4] that OHHs were minimally reported by healthcare professional in many developing countries. In light of this, there is the need to intensify efforts in institutionalizing guidelines for reporting exposure to occupational hazards in psychiatric hospitals in the country.

Also, there is the need to focus some attention to non-professional cadre of frontline workers in psychiatric hospitals although the differences in risk of exposure to OHHs among the professional categories were not statistically significant. In Ghana, non-professional nursing staff are not given comprehensive training in mental health nursing during their pre-service education as compared to the professional registered mental health nurses. This pre-service training gap could be a recipe for higher risk of exposure to OHHs among these non-professional groups although not substantiated in this study. Nonetheless, it is recommended that continuous professional development (CPDs) on safety precautions should be initiated for these cadres of staff to help enhance their knowledge/skills on occupational health and safety. This intervention could help to eventually reduce their chances of exposure to OHHs in the work environment.

Additionally, it was found that frontline staff who practised for many years on the ward did not necessarily have lower chance of exposure to OHHs, contrary to similar previous studies [8–10] outside Ghana. Rather, more experienced staff were more likely to be exposed to OHHs than their relatively in-experienced colleagues. Even though no available relevant literature was found to corroborate this finding, it is possible staff who spent more years working on the ward overtime took the safety conditions for granted due to familiarity with the work environment hence increasing their likelihood of exposure to the OHHs.

Overall, it was found that safety conditions in the two psychiatric hospitals were poor as per the views of the frontline nursing staff. These perceived poor safety conditions do not only constitute an important disincentive but also potentially affect the quality of healthcare delivery to psychiatric patients. Limited resources/logistics, lack of emergency exit points, and security were particularly emphasized by many respondents as major safety threats to frontline health staff in psychiatric hospitals in Ghana. These findings depict concerns raised in similar previous studies on occupational health and safety in sub-Saharan Africa [35–39].

Moreover, it is important to emphasize that the findings of this study vis-à-vis other previous studies on occupational health and safety might bear variations because of the different cultural, social and political dynamics in the developed and under-developed countries. For instance, in developed healthcare systems due to the availability of physical infrastructure and absence of crowding in hospital wards, physical assaults and related health hazards are recorded minimally. Conversely, in Ghana like other developing countries, limited health infrastructure resulting in overcrowding induce frontline staff exposure to OHHs in psychiatric hospital environments.

## Conclusions

Based on the study findings, it is concluded that the work of frontline nursing staff working in psychiatric hospitals in Ghana is risky and potentially unsafe given the poor health infrastructure situation in these health facilities. The situation is aggravated by the lack of clear reporting system for OHHs, documentation and seeking redress for victims of work related health hazards. Limited knowledge of health staff on what constitutes health hazards also potentially clouds true incidence and prevalence rates of these OHHs, particularly non-physical abuses which are often under-estimated and hence under-reported.

It is therefore compelling to institute policy reforms in the health sector especially in psychiatric hospitals. With the establishment of the Mental Health Authority Act (846, 2012) [40], it is expected efforts will be intensified on the health and safety of frontline health workers in psychiatric hospitals. There is the need to institute or revamp existing occupational health and safety committees in psychiatric hospitals in Ghana to monitor, report and help enforce policies on occupational health and safety at the health facility levels. It is also recommended that the Workman Compensation Act of 1987 (PNDCL 187) [37] be amended and streamlined to meet the specific contemporary needs of the psychiatric hospitals. Furthermore, monthly training and education sessions should be instituted and sustained for frontline health workers on psychosocial OHHs and their prevention thereof. Finally, standardized procedures for reporting work related health hazards should be established and clinical and administrative health workers educated on these procedures.

## Additional file

Additional file 1: Questionnaire. Content description of the structured questionnaire used for the data collection. (PDF 72 kb)

## Abbreviations

APH: Accra Psychiatric Hospital
GAR: Greater Accra Region
GHS: Ghana Health Service
HAs: Health Aids
HEW: Health Extension Workers
ILO: International Labour Organization
JCOHS: Joint Committee on Occupational Health and Safety
MOH: Ministry of Health
NAC: Nurse Assistants Clinical
NAP: Nurse Assistants Preventative
OHHs: Occupational Health Hazards
OHS: Occupational Health and Safety
OPD: Outpatient Department
PPH: Pantang Psychiatric Hospital
SPSS: Statistical Package for Social Sciences
WHO: World Health Organization
WO: Ward Orderlies

## Footnotes

1 Psychiatric health workers: nurses and nurse-assistants providing direct health care to patients in the two sampled psychiatric hospitals. Nurses: health professionals trained as a registered general nurse, registered psychiatric nurse, community psychiatric nurse or any other category under the nursing fraternity Nurse-assistant: a health worker trained to assist the professional nurse in the clinical setting. They include nurse assistant clinical (NAC), nurse assistant preventive (NAP), word orderlies, health extension workers Key informants: individuals in managerial or administrative positions who can influence policy decisions and formulations in the two sampled hospitals. Psychiatric health care facility: health institution specialized in psychiatric healthcare for patients with mental conditions. Health hazard: psychological, physical, biological, ergonomical and chemical agents and factors that have the potential of causing a health related problem when one is exposed to them. Physical health hazard: factors, events or situations present in a work environment that have the potential to compromise the physical and psychosocial wellbeing of workers (e.g. physical assault, battery). Psychological health hazard: emotional events or situations present in a workplace that have the potential to compromise the physical and psychosocial wellbeing of workers (verbal abuse, work related stress, superior relations with subordinates). Biological health hazard: a category of health hazards that involve exposure to any form of viral, bacterial or fungal infections in the course of performing one’s duties. Workplace: the two sampled psychiatric hospitals where healthcare workers give care to mental health patients.
