# Supplementary material for: Experiences of frontline nursing staff on workplace safety and occupational health hazards in two psychiatric hospitals in Ghana
Source: BMC Public Health. 2018 Jun 6;18:701. doi: 10.1186/s12889-018-5620-5 (PMC5989379; doi:10.1186/s12889-018-5620-5)
Supplement: Supplementary file 1 — Questionnaire. Content description of the structured questionnaire used for the data collection. (PDF 72 kb) [file 12889_2018_5620_MOESM1_ESM.pdf]

<u>Appendix 1:</u> Questionnaire for frontline nursing staff working in psychiatric hospitals

Research Topic: Occupational Health and Safety in Psychiatric Hospitals in Ghana.

This questionnaire is a data collection instrument designed by the researchers to gather information on occupational health and safety in selected mental health hospitals. You are kindly requested to answer the questions as they apply to you, by checking the boxes or filling in the spaces provided. The study is purely for academic purposes and your confidentiality is guaranteed. By ticking the "Yes Agree" box you confirm to voluntarily participate in this research. Nonetheless, you are always at liberty to exit at any point in time from the study without any consequences regarding your career here or your persona life.

Thanks for your time. For details of the research contact the lead research on Mobile number: 0241226409 or email: arkabason@yahoo.com .

Do you confirm to have understood the purpose of the study and voluntarily agreed to particupate in this study?

Yes Agree ☐ *No Disagree* ☐

Please give reasons if can for choosing not to participate……………………………………………………….

Questionnaire No.:…………. Date Given:………. Date Received………

SECTION A: RESPONDENT'S BIOGRAPHIC DATA	RESPONSE
Sex	Male ☐ Female ☐
Age	Please specify:………………………………

SECTION B: RESPONDENT'S WORK HISTORY	RESPONSE
Employment contract	Permanent staff ☐<br>Part time staff ☐<br>Other (specify)…………………………..
Ward/unit of work	Please specify……………………………….

Professional category	Registered Psychiatric Nurse ☐<br>Registered General Nurse ☐<br>Enrolled Nurse ☐<br>Community Psychiatric Nurse ☐<br>Health Assistant Clinical ☐<br>Health Extension Worker ☐<br>Ward Orderly ☐<br>Health Aid ☐<br>Other (specify)…………………………….
Ward/unit of work	Please specify……………………………….
How long have you been working in this health facility?	Less than a year ☐<br>One year ☐<br>Two years ☐<br>Three years ☐<br>Four years ☐<br>Five years ☐<br>More than five years ☐

SECTION C: RESPONDENT'S KNOWLEDGE AND SOURCES OF INFORMATION ON OCCUPATIONAL HEALTH AND SAFETY (OHS)	RESPONSE
1. Have you ever heard of occupational health and safety in the health care sector?	Yes ☐<br>No ☐
2. If **yes** to question 1 which one of these were the predominant source of information to you on OHS?<br><br>**NOTE**: Multiple responses not allowed	Through the media ☐<br>Through a friend ☐<br>Through a co-worker ☐<br>Through a seminar/workshop ☐<br>Other (Please specify one)…..……………….
3. Do you think your work in the psychiatric hospital is risky in any way than other healthcare facilities?	Yes ☐<br>No ☐
4. If yes to question 3 what are the five key health risks/hazards associated with your work?	1………………………………………..<br>2………………………………………..<br>3………………………………………..<br>4………………………………………..<br>5………………………………………..
**SECTION D: RESPONDENT'S EXPERIENCE OF OCCUPATIONAL HEALTH HAZARDS**	**RESPONSE**
5. Have you ever been exposed to any form of health hazard(s) in the course of performing your duties in this hospital?	Yes ☐<br>No ☐

6. If **yes** to question 5 which one of these were you exposed to most?<br><br>**NOTE:** Multiple responses not allowed	Biological Health Hazards ☐<br>Physical Health Hazards ☐<br>Psychosocial Health Hazards ☐
7. How many times do you experience occupational health hazards on daily basis in this hospital?	One-four times ☐<br>Five times ☐<br>More than five times ☐<br>Ten times ☐<br>More than ten times ☐<br>Cannot remember ☐
8. In the past one year, how many times were you exposed to occupational health hazards in this hospital?	Once ☐<br>Five times ☐<br>More than five times ☐<br>Ten times ☐<br>More than ten times ☐<br>Cannot remember ☐

9. Which of these physical health hazards do you experience most in this health facility? (You can check more than one option)	Back injury due to patient lifting ☐<br>Physical violence from patients ☐<br>Physical violence from patient's relatives ☐<br>Needle prick ☐<br>Falls on slippery floor ☐<br>Direct skin contact of patient's body fluids (blood, urine, saliva, faeces etc.) ☐<br>Other (specify)………………………………
10. Which of these psychosocial health hazards do you experience most as a health worker in this hospital? (You can check more than one option)	Unfavorable work time schedules (work shift) ☐<br>Work load and demand ☐<br>Unrealistic expectations from patients ☐<br>Verbal abuse and threats from patients ☐<br>Unrealistic expectations from superiors ☐<br>Frustrations due to limited resources ☐<br>Low morale/poor organizational climate ☐<br>Other (specify)……………………..
11. Have you ever been diagnosed of work related medical condition (s)?	Yes ☐<br>No ☐
12. If yes to question 11, please specify the name of medical condition (s)	Please specify…………………………

SECTION E: RESPONSIBILITY FOR WORK RELATED HEALTH HAZARDS	RESPONSE
13. How often do you think your exposure to an occupational health hazard is due to your personal negligence?	None of the occasions ☐<br>Once in ten occasions ☐<br>Twice in ten occasions ☐<br>Most of the occasions ☐<br>On all occasions ☐
14. How often do you think your exposure to an occupational health hazard is due to system failure (Hospital management fault) or co-workers' negligence?	None of the occasions ☐<br>Once in ten occasions ☐<br>Twice in ten occasions ☐<br>Most of the occasions ☐<br>On all occasions ☐

SECTION F: RESPONDENT'S REPORTING SYSTEM OF EXPOSURE TO OCCUPATIONAL HEALTH HAZARDS	RESPONSE
15. Have you ever reported an exposure to occupational health hazard (s) to hospital management?	Yes ☐<br>No ☐
16. If **yes** to question 15 what type of occupational health hazard (s) was it or were they?	Please specify…………………………
17. Which of these factors influenced you to report the occupational health hazard (s) exposed to?	Perceived degree of severity of health hazard ☐<br>Co-worker influence ☐<br>The easy reporting system ☐<br>Other (please specify).............................
18. If **no** to question 15 please give **four main** reasons why you never reported an exposure to occupational health hazard (s) to hospital management	1………………………………………<br>2……………………………………..<br>3……………………………………<br>4……………………………………..

SECTION G: RESPONDENT’S PERCEPTION OF SAFETY CONDITIONS OF PSYCHIATRIC HOSPITAL					
**Please circle how you will describe the general occupational health and safety conditions of your health facility**	**VERY GOOD 5**	**GOOD 4**	**AVERAGE 3**	**BAD 2**	**VERY BAD 1**
**Perception of safety conditions in the hospital**					
19. Availability of logistics and material resources in the wards	5	4	3	2	1
20. State of emergency exit points in the hospital	5	4	3	2	1
21. Security measures for workers against patients attacks	5	4	3	2	1
22. State of floors in the wards and hospital	5	4	3	2	1
23. Procedures for reporting exposure to occupational health hazards in the hospital	5	4	3	2	1
24. Contribution of hospital management towards reducing incidence of occupational health hazards in the	5	4	3	2	1
25. Preparedness of the hospital for any unforeseen disaster	5	4	3	2	1
26. Availability of preventive measures for occupational health hazards in the hospital	5	4	3	2	1
27. Compensation packages for health workers who get exposed to occupational health hazards in the course of their duties	5	4	3	2	1

SECTION H: SUGGESTIONS FOR IMPROVING HEALTH AND SAFETY OF HEALTH WORKERS IN PSYCHIATRIC HOSPITAL

28. Do you have suggestions for improvement of the health and safety of staff in this hospital? Yes _______ No______

29. If yes, to question 28 please specify your suggestions to hospital management?………….

30. What do you think health workers in psychiatric hospitals need to do to ensure their personal health safety in the course of their duties?...

31. Which areas do you think health personnel in psychiatric hospitals need training and education on occupational health and safety?..

Thanks for Your Cooperation
